# Beyond HELLP: An Unusual Cause of Severe Thrombocytopenia in Pregnancy

**DOI:** 10.7759/cureus.103731

**Published:** 2026-02-16

**Authors:** Rinchen Zangmo, Geetha Mahindrakar, Rajesh Utterkar

**Affiliations:** 1 Obstetrics and Gynaecology, Gwynedd Hospital, Betsi Cadwaladr University Health Board, Bangor, GBR; 2 Anaesthesiology, Gwynedd Hospital, Betsi Cadwaladr University Health Board, Bangor, GBR

**Keywords:** hellp syndrome, immune thrombocytopenic purpura, preeclampsia, thrombocytopenia, thrombotic thrombocytopenic purpura

## Abstract

Thrombotic thrombocytopenic purpura (TTP) is a rare yet potentially fatal thrombotic microangiopathy that poses significant risks during pregnancy. It is characterized by microangiopathic hemolytic anemia, profound thrombocytopenia, and widespread microvascular thrombosis. We present a case of pregnancy-associated TTP presenting with isolated severe thrombocytopenia, highlighting the diagnostic challenge. A 27-year-old, G3P1 woman at 31+6 weeks of gestation presented with hypogastric pain and a resolved episode of vomiting. Laboratory evaluation revealed severe thrombocytopenia (platelets: 9 × 10⁹/L), mild anemia, and proteinuria. Differential diagnosis included immune thrombocytopenic purpura (ITP), HELLP (hemolysis, elevated liver enzymes, and low platelet count), and TTP. Initial therapy included corticosteroids, platelet transfusions, primarily suspecting ITP as the first differential diagnosis, and magnesium sulfate later after lactate dehydrogenase levels were suggestive of hemolysis. HELLP syndrome being the main differential, counseling was done about the possible need for preterm delivery, and a neonatologist was involved for discussion with the parents. This was followed by urgent ADAMTS13 testing, which confirmed TTP. The patient was transferred to a tertiary care center for plasma exchange therapy, which improved the platelet count. She subsequently underwent induction of labor at 35 weeks for severe preeclampsia, delivering vaginally with postpartum hemorrhage (1.7 L) managed with transfusions. Both maternal and neonatal outcomes were favorable, and both were discharged on day 10 of delivery. This case illustrates the atypical presentation of TTP, where severe thrombocytopenia may occur without classic neurological or renal symptoms. Timely recognition of the correct diagnosis allowed appropriate treatment and prevented unnecessary very preterm delivery.

## Introduction

Thrombotic thrombocytopenic purpura (TTP) is a rare thrombotic microangiopathy characterized by microangiopathic hemolytic anemia, severe thrombocytopenia, and ischemic end-organ injury due to microvascular platelet-rich thrombi with pathological features including occlusive microvascular disease [1-3]. It is caused by a severe deficiency of the metalloproteinase ADAMTS13 [4]. The condition predominantly affects women of reproductive age, with pregnancy serving as a major triggering factor in approximately 12-25% of adult-onset cases [5]. TTP can resemble other pregnancy-associated disorders, including HELLP syndrome (hemolysis, elevated liver enzymes, and low platelet count) and preeclampsia, but it requires distinct treatment strategies. Prompt and accurate diagnosis is critical, as TTP is treated with therapeutic plasma exchange and immunosuppression, whereas HELLP syndrome is typically managed by delivery of the pregnancy [5]. We present this case to highlight the diagnostic challenge of an unusual presentation of severe thrombocytopenia in pregnancy and its implications on maternal and fetal outcomes.

## Case presentation

A 27-year-old G3P1 woman at 31+6 weeks presented with hypogastric pain and a recent self-limited episode of vomiting. The pain began a day earlier; worsened with movement, eating, and drinking; and followed a week of nausea and vomiting. She had no headache, visual disturbance, fever, rash, bleeding, bruising, vaginal loss, or pelvic pain, and was not on anticoagulants.

Her obstetric history included a first pregnancy complicated by gestational diabetes at 27 weeks and resulting in a spontaneous preterm vaginal delivery at 36+4 with no maternal or neonatal complications. This was followed by a missed miscarriage at eight weeks in November 2024, managed surgically. In the current pregnancy, she was consultant-led due to a body mass index of 40.6 kg/m². Prenatal blood tests performed at the time of booking were normal with a hemoglobin of 120 g/L and platelet count of 220 × 10⁹/L. Her glucose tolerance test at 28 weeks was normal, and fetal growth was appropriate.

**Figure 1:**
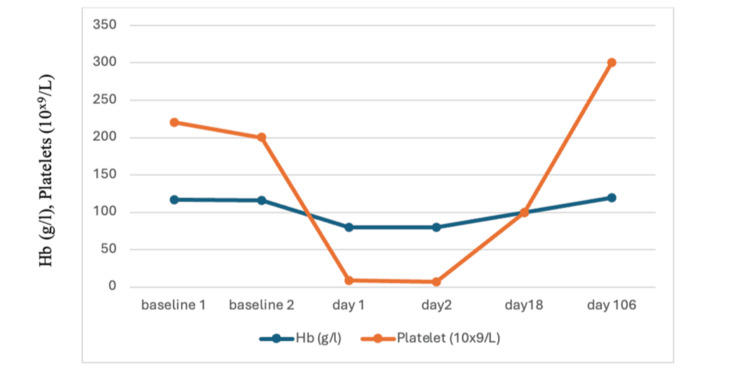
Hemoglobin (Hb) and platelet count trend.

During the current admission, her observations were normal, including a heart rate of 62 beats/minute, blood pressure of 119/78 mmHg, temperature of 37°C, and normal deep tendon reflexes. Her abdomen was soft with mild hypogastric tenderness, and the uterus was relaxed and non-tender. Repeat investigations showed a drop in hemoglobin and a significant drop in platelet count (Table 1) with normal white cell count, amylase, renal function, liver enzymes, and coagulation profile. Bilirubin was elevated, urine dipstick showed +++ protein, and the urine protein-creatinine ratio was raised. Cadiotocography (CTG) for fetal monitoring was normal. A repeat full blood count (FBC) showed a further drop in platelet count, as shown in Table 1 and Figure 1.

**Table 1:** Laboratory investigations.

Investigations	Baseline	Day 1	Day 2	Day 18	Day 106	Reference range
Hemoglobin	120	86	83	100	119	115–165 g/L
White cell count	6.0	6.2	6.9	7.0	6.9	4.0–11.0 × 10⁹/L
Platelet count	220	9	7	100	300	150–400 × 10⁹/L
Reticulocyte count	70	102	101	-	-	50–100 × 10⁹/L
Lactate dehydrogenase		557	-	-	-	135–225 IU/L
Serum amylase		94				30–110 IU/L
Prothrombin time	-	11	-	11.4	-	10–14 seconds
Activated partial thromboplastin time	-	24	-	23	-	25–35 seconds
Urea	-	5.0	-	5.9	-	2.5–7.8 mmol/L
Creatinine	-	58	-	60	-	49–90 µmol/L
Bilirubin	-	32	-	19	-	3–21 µmol/L
Alanine aminotransferase	-	30	-	37	-	<40 IU/L
Aspartate aminotransferase	-	25	-	36	-	<40 IU/L
Albumin	-	29	-	27	-	35–50 g/L
Globulin	-	20	-	23	-	20–35 g/L)
Urine protein-creatinine ratio	-	425	-	-	-	<30 mg/mmol

Hematology was consulted, and the differential diagnosis included immune thrombocytopenic purpura (ITP), TTP, and HELLP syndrome. She was started on prednisolone 50 mg daily and transfused one unit of platelets, considering ITP as the first differential diagnosis. Further tests showed raised lactate dehydrogenase (LDH) and an increased reticulocyte count, indicating hemolysis and shifting the diagnostic focus to HELLP syndrome. Magnesium sulfate was commenced for suspected atypical HELLP, and intravenous dexamethasone was administered for fetal lung maturity. A multidisciplinary discussion was held regarding maternal monitoring, the potential need for general anesthesia and autologous blood transfusion, and whether transfer to a tertiary center was advisable.

Placental growth factor (PlGF) returned amber (56 pg/mL), less suggestive of immediate risk, and blood pressure remained normal throughout, making pre-eclampsia less likely. With increasing suspicion of TTP, ADAMTS13 testing was arranged urgently via a tertiary center. The result showed severely reduced ADAMTS13 activity at 7%, confirming TTP. Magnesium sulfate was stopped, and she was transferred immediately for plasma exchange therapy. Her observations and fetal monitoring were normal at the time of transfer. Her platelet count increased to 100 × 10⁹/L after 10 days of treatment.

At 34 weeks, she developed hypertension and was commenced on labetalol 200 mg three times daily. Labor was induced at 35 weeks due to worsening proteinuria and pre-eclampsia. She delivered vaginally eight hours later, complicated by a second-degree perineal tear and a postpartum hemorrhage of 1.7 L, requiring two units of blood and intravenous ferric derisomaltose. She did not require further plasma exchange and was discharged with her baby on day 10 postpartum. Her blood counts continued to improve, and by three months postpartum, her platelet count had increased to 300 × 10⁹/L (Figure 1 and Table 1).

## Discussion

TTP is a rare but life-threatening emergency driven by a marked deficiency of the metalloproteinase ADAMTS13 [1]. It normally cleaves von Willebrand factor multimers, but in TTP, its deficiency leads to abnormal platelet aggregation and widespread microvascular thrombi formation, causing organ damage, hemolytic anemia, and thrombocytopenia. This results in impaired blood flow and significant end-organ perfusion issues [1,6,7].

Pregnancy can trigger TTP due to physiological changes in coagulation, significantly increasing the risk when ADAMTS13 levels are severely deficient [8]. Symptoms of TTP range from asymptomatic to severe, often mimicking other pregnancy-related complications such as HELLP syndrome and preeclampsia, making diagnosis challenging. Clinical signs include thrombocytopenia, hemolytic anaemia, neurological deficits, fever, and renal dysfunction, with fetal loss and fetal growth restriction also being common [8].

There are a limited number of case reports on TTP in pregnancy. There was one case of a pregnant woman at 26th week with a sudden onset of left-hand paraesthesia and purpura. Diagnosis of TTP was made based on the low ADAMTS13 level. She was also found to be human immunodeficiency virus positive on admission. Plasmapheresis and antiretroviral therapy were initiated, and the delivery of a healthy newborn at full-term gestation was achieved, with no obstetric complications [9].

Andreatidis et al. reported the case of a 28-year-old woman at 34 weeks of gestation found to have thrombocytopenia (platelets: 23 × 10⁹/L) and normocytic anemia (hemoglobin: 89 g/L) during her third pregnancy on routine testing. She had quiescent systemic lupus erythematosus on prednisolone, hydroxychloroquine, and azathioprine, and a small-for-gestational-age fetus. Examination was unremarkable. Investigations showed creatinine 0.57 mg/dL, LDH of 357 U/L, mild schistocytes, and a normal sFlt-1/PlGF ratio. ADAMTS13 activity was reduced, confirming acquired TTP. Steroid escalation was ineffective; daily plasma exchange improved platelets to 140 × 10⁹/L, then falling to 103 × 10⁹/L. Labor was induced at 36 weeks, delivering a healthy 2,082 g infant. Postpartum, the mother received weekly rituximab (375 mg/m²). The newborn required 30 days of special-care admission for prematurity, transient tachypnea, and hypoglycemia [10].

Dap et al. reported the case of a 28-year-old woman who developed severe thrombocytopenia (platelets: 34 → 6 × 10⁹/L), new anemia, and rising aspartate aminotransaminase (91 → 116 U/L) at 31 weeks. Plasmapheresis was ineffective. She then developed hypertension, proteinuria of 15 g/24 hours, and worsening liver enzymes, prompting a cesarean section on day four for suspected HELLP syndrome. Postpartum, platelets and liver tests improved. The sFlt-1/PlGF ratio was 855, ADAMTS13 activity was <5%, and an *ADAMTS13* gene mutation was identified, confirming TTP. The case showed that TTP and HELLP can coexist and exacerbate each other, with TTP contributing to placental malperfusion [11].

In their retrospective multicenter study, Béranger et al. [12] highlighted the challenges associated with pregnancy-onset TTP and childbirth in terms of diagnosis, obstetric management, and follow-up. Among 1,174 pregnancy-onset thrombotic microangiopathies (TMAs) enrolled in the French Registry for TMA from 2000 to 2020, 108 pregnancy-onset TTP were identified. The primary ethnic backgrounds represented were Caucasian (63.8%), Afro-Caribbean (19.4%), and North African (14.8%). When present, notable associated contexts included systemic autoimmune diseases (16.7%), infections (15.7%), ovarian stimulation for in vitro fertilization (4.6%), and recent abortion (1.9%). The median age was 28 years. The median gestational age at the TTP episode was 30 weeks. Maternal survival rate was 95%, with 103 of 108 women surviving. Common presenting features included fever (25.9%), neurological manifestations (46.3%), hypertension (26.8%), abdominal pain (38.9%), and acute kidney injury (40.7%). The median platelet count was 18 × 10⁹/L (interquartile range: 20 × 10⁹/L). Fetal survival rate was 45.5% in the first trimester, 24% in the second, and 90.6% in the third trimester. All available placental histology described marked placental ischemia with numerous intervillous thrombi of varying ages. This large multicenter study showed that pregnancy-onset TTP is rare but serious, with diagnosis often difficult because it presents across all trimesters and overlaps clinically with other pregnancy-related TMAs. Maternal survival is high (95%) with appropriate treatment, but fetal outcomes depend heavily on gestational age at TTP onset, with very poor survival in the first and second trimesters and much better outcomes in the third trimester. The study also highlights that most cases occur without an identifiable trigger, though autoimmune disease, infection, and ovarian stimulation are notable associations. Placental pathology confirms that TTP profoundly impairs placental perfusion, contributing to fetal loss.

## Conclusions

This case underscores an atypical presentation of TTP in pregnancy, demonstrating that severe and rapidly progressive thrombocytopenia may occur in the absence of the classical neurological or renal manifestations. Such presentations pose a significant diagnostic challenge, particularly in the context of pregnancy, where overlapping features with hypertensive and microangiopathic disorders are common. Early clinical suspicion, prompt initiation of ADAMTS13 activity testing, and close collaboration between obstetric, hematology, and critical care teams are crucial to facilitating timely diagnosis and intervention. Accurate differentiation between TTP and HELLP syndrome is critical, as misdiagnosis may result in inappropriate management, including unnecessary iatrogenic preterm delivery, without addressing the underlying pathology. In contrast, correct and timely identification of TTP enables early initiation of plasma exchange therapy, which is associated with substantial reductions in maternal morbidity and mortality and improved perinatal outcomes. This case highlights the need for heightened awareness of atypical TTP presentations in pregnancy and reinforces the importance of a multidisciplinary approach to optimize both maternal and fetal outcomes.

## References

[1] Sukumar S, Lämmle B, Cataland SR (2021). Thrombotic thrombocytopenic purpura: pathophysiology, diagnosis, and management. J Clin Med.

[2] Cuker A, Cataland SR, Coppo P (2021). Redefining outcomes in immune TTP: an international working group consensus report. Blood.

[3] Scully M, Rayment R, Clark A (2023). A British Society for Haematology Guideline: diagnosis and management of thrombotic thrombocytopenic purpura and thrombotic microangiopathies. Br J Haematol.

[4] Sadler JE (2008). Von Willebrand factor, ADAMTS13, and thrombotic thrombocytopenic purpura. Blood.

[5] Golsorkhi M, Ebrahimi N, Kordasti S, Norouzi S, Abdipour A (2025). Navigating recurrent immune-mediated thrombotic thrombocytopenic purpura (iTTP) in pregnancy: A case report and literature review. Transfus Apher Sci.

[6] Furlan M, Robles R, Lämmle B (1996). Partial purification and characterization of a protease from human plasma cleaving von Willebrand factor to fragments produced by in vivo proteolysis. Blood.

[7] Gao W, Anderson PJ, Sadler JE (2008). Extensive contacts between ADAMTS13 exosites and von Willebrand factor domain A2 contribute to substrate specificity. Blood.

[8] Sundin C, Rhodes K (2025). Thrombotic thrombocytopenic purpura in pregnancy. MCN Am J Matern Child Nurs.

[9] Marins LR, da Rocha Oppermann ML (2021). Thrombotic thrombocytopenic purpura and acquired immunodeficiency syndrome diagnosed in pregnancy: case report. J Obstet Gynaecol Res.

[10] Andreatidis B, Weber N, Craven AM, Duke M, Callaway L, Eccles-Smith J (2025). Thrombotic thrombocytopenic purpura in pregnancy: a case report and literature review. Obstet Med.

[11] Dap M, Romiti J, Dolenc B, Morel O (2022). Thrombotic thrombocytopenic purpura and severe preeclampsia: a clinical overlap during pregnancy and a possible coexistence. J Gynecol Obstet Hum Reprod.

[12] Béranger N, Coppo P, Tsatsaris V (2024). Management and follow-up of pregnancy-onset thrombotic thrombocytopenic purpura: the French experience. Blood Adv.

